# A road map for leptospirosis research and health policies based on country needs in Latin America

**DOI:** 10.26633/RPSP.2017.131

**Published:** 2017-10-24

**Authors:** Martha Maria Pereira, Maria Cristina Schneider, Claudia Munoz-Zanzi, Federico Costa, Jackie Benschop, Rudy Hartskeerl, Julio Martinez, Michel Jancloes, Eric Bertherat

**Affiliations:** 1 Instituto Oswaldo Cruz WHO Collaborating Center for Leptospirosis Rio de Janeiro Brazil Instituto Oswaldo Cruz, WHO Collaborating Center for Leptospirosis, Rio de Janeiro, Rio de Janeiro, Brazil.; 2 Pan American Health Organization Health Emergencies Department, Washington, D.C. United States of America Pan American Health Organization, Health Emergencies Department, Washington, D.C., United States of America.; 3 University of Minnesota Twin Cities Minneapolis Minnesota United States of America University of Minnesota Twin Cities, Minneapolis, Minnesota, United States of America.; 4 Instituto de Saúde Coletiva Universidade Federal da Bahia SalvadorBahia Brazil Instituto de Saúde Coletiva, Universidade Federal da Bahia, Salvador, Bahia, Brazil.; 5 Massey University, Institute of Veterinary Animal and Biomedical Sciences Molecular Epidemiology and Public Health Laboratory Palmerston North New Zealand Massey University, Institute of Veterinary Animal and Biomedical Sciences, Molecular Epidemiology and Public Health Laboratory, Palmerston North, New Zealand.; 6 WHO/FAO/OIE and National Leptospirosis Reference Centre WHO/FAO/OIE and National Leptospirosis Reference Centre Amsterdam Netherlands WHO/FAO/OIE and National Leptospirosis Reference Centre, Amsterdam, Netherlands.; 7 Health and Climate Foundation Health and Climate Foundation Marchissy Switzerland Health and Climate Foundation, Marchissy, Switzerland.; 8 World Health Organization World Health Organization Geneva Switzerland World Health Organization, Geneva, Switzerland.

**Keywords:** Leptospirosis, health planning guidelines, health services research, Latin America, Leptospirosis, directrices para la planificación en salud, investigación en servicios de salud, América Latina, Leptospirose, diretrizes para o planejamento em saúde, pesquisa sobre serviços de saúde, América Latina

## Abstract

*This report summarizes the presentations, discussions and the recommendations coming from the Oswaldo Cruz Institute/FIOCRUZ International Workshop for Leptospirosis Research Based on Country Needs and the 5th Global Leptospirosis Environmental Action Network meeting, which was held in the city of Rio de Janeiro, Brazil, 10–12 November 2015. The event focused on health policy and worked to develop a road map as a consensus document to help guide decision-making by policymakers, funding bodies, and health care professionals. The direction that leptospirosis research should take in the coming years was emphasized, taking into account the needs of countries of Latin America, as well as experiences from other world regions, as provided by international experts. The operational concepts of “One Health” and translational research underlaid the discussions and the resulting recommendations. Despite the wide geographic distribution of leptospirosis and its impact in terms of incidence, morbidity, and mortality, leptospirosis is not yet considered a “tool-ready” disease for global initiatives. Surveillance programs need new tools and strategies for early detection, prevention, and follow-up. The major recommendations developed at the Rio meeting cover both health policy and research. The health policy recommendations should be taken into account by decisionmakers, government officials, and the Pan American Health Organization. The priorities for research, technological development, and innovation should be considered by research institutions, universities, and stakeholders*.

## RESUMEN

En este informe se resumen las ponencias, los debates y las recomendaciones del taller internacional para la investigación sobre la leptospirosis basada en las necesidades de los países organizado por el Instituto Oswaldo Cruz/FIOCRUZ y la quinta reunión de la Red Global de Acción Ambiental contra la Leptospirosis, que se celebró en la ciudad del Rio de Janeiro (Brasil), del 10 al 12 de noviembre del 2015. El evento se centró en las políticas de salud y en la elaboración de una hoja de ruta que sirviese como un documento guía consensuado para la toma de decisiones de los formuladores de políticas, los organismos de financiamiento y los profesionales de la salud. Se hizo hincapié en la dirección que tiene que tomar la investigación sobre la leptospirosis en los próximos años, en la que se deben tener en cuenta las necesidades de los países de América Latina, así como a las experiencias de otras regiones del mundo, de acuerdo con las consideraciones de los expertos internacionales. Los conceptos operativos de “Una sola salud” y de la investigación traslacional fueron la base de los debates y de las recomendaciones resultantes. A pesar de la extensa distribución geográfica de la leptospirosis y su repercusión desde el punto de vista de la incidencia, la morbilidad y la mortalidad, la leptospirosis no se considera todavía una enfermedad con herramientas listas para ser aplicadas (tool-ready) en las iniciativas a nivel mundial. Los programas de vigilancia necesitan herramientas y estrategias nuevas para llevar a cabo la detección temprana, la prevención y el seguimiento. Las recomendaciones principales que se emitieron en la reunión de Rio cubrieron tanto el ámbito de las políticas de salud como el de la investigación. Las recomendaciones que se refieren a las políticas de salud deben ser tenidas en cuenta por los encargados de tomar decisiones, los funcionarios de gobierno y la Organización Panamericana de la Salud. Las prioridades de la investigación, el desarrollo tecnológico y la innovación deben ser incorporadas por las instituciones de investigación, las universidades y los interesados directos.

## RESUMO

Trata-se de um resumo das apresentações, debates e recomendações do Workshop Internacional para Pesquisa em Leptospirose com Base nas Necessidades dos Países do Instituto Oswaldo Cruz/FIOCRUZ e 5a Reunião da Rede Global de Ação Ambiental em Leptospirose, realizados na cidade do Rio de Janeiro, Brasil, de 10 a 12 de novembro de 2015. Os participantes do evento debateram políticas de saúde e elaboraram um guia como documento de consenso para orientar a tomada de decisão pelos responsáveis por políticas, agências de financiamento e profissionais da saúde. Destacou-se o rumo a ser tomado em pesquisa em leptospirose nos próximos anos levando em consideração as necessidades dos países da América Latina, assim como as experiências de outras regiões do mundo, conforme estabelecido por especialistas internacionais. Os conceitos práticos da perspectiva “One Health” (Um Mundo, Uma Saúde) e pesquisa translacional foram a base dos debates e das recomendações conseguintes. Apesar da ampla distribuição geográfica e do seu impacto em termos de incidência, morbidade e mortalidade, a leptospirose ainda não é considerada uma doença “tool ready” para iniciativas globais. Os programas de vigilância precisam de novas ferramentas e estratégias para detecção precoce, prevenção e acompanhamento. As principais recomendações elaboradas na reunião do Rio englobam política de saúde assim como pesquisa e elas devem ser levadas em consideração pelas autoridades, funcionários do governo e Organização Pan-Americana da Saúde. As instituições de pesquisa, universidades e interessados diretos devem atentar para as prioridades em pesquisa, desenvolvimento tecnológico e inovação.

Leptospirosis is a disease that has a significant health impact in various regions of the world and that affects people from different socioeconomic levels. This zoonotic disease of epidemic potential has been causing outbreaks for many years, mainly related to heavy rain events in geographic areas prone to flooding (1–6). Leptospirosis is also considered a neglected disease that is associated with populations living in vulnerable conditions in urban and rural settings. Many leptospirosis cases are related to specific occupations, such as farming and handling animals (7–10). Despite the increasing number of cases and outbreaks globally, leptospirosis remains relatively unrecognized (11, 12).

Leptospirosis in humans is associated with a high number of cases, as well as high morbidity and fatality rates. Concerns about animal leptospirosis are related to its impact on animal health, to economic losses, and to risk situations in the human-animal-ecosystem interface. Even with the progress made by researchers and government authorities, there are still important knowledge gaps that hinder both surveillance efforts and prevention and control activities.

One step toward filling those knowledge gaps came with the International Workshop of the Oswaldo Cruz Institute/FIOCRUZ for Leptospirosis Research Based on Country Needs and 5th Global Leptospirosis Environmental Action Network (GLEAN) meeting, which was held in the city of Rio de Janeiro, Brazil, 10-12 November 2015. The meeting focused on health policy, with the objective of developing a road map for leptospirosis research based on country needs. The 50 attendees included policymakers, health professionals, and researchers from the Americas, Europe, Asia, and Africa, as well as New Zealand. The discussions were comprehensive, involving experts from multiple disciplines, with noteworthy contributions on specific topics.

## Novel aspects of the meeting

The main purpose of the meeting was to find ways to change the current situation of leptospirosis from a disease that is neglected, but not listed as such by the World Health Organization (WHO), into a “tool-ready” disease for global initiatives. The meeting efforts to assess country needs and the state of the art with respect to leptospirosis were mainly focused on Latin America, but health professionals, decisionmakers, and researchers from other world regions also contributed with their knowledge and experiences. This integrated approach in a highly specialized international forum led to extensive discussions and to results that may represent a turning point for developments in the area of leptospirosis in the near future.

## Leptospirosis as a “tool-ready” disease

An infectious disease that can be controlled or even eliminated through mass administration of safe and effective treatment or other effective interventions, such as vaccines, is considered to be a “tool-ready” disease.

A disease that does not yet have easy tools for interventions is more difficult for decision makers to select as a priority for government agendas, even if its prevalence is high, due to the uncertainty in the outcome of a systematic or large-scale control program.

WHO and its partners developed a road map for neglected tropical diseases that are “tool-ready” (13, 14). Unfortunately, leptospirosis is not targeted in that initiative. Leptospirosis could be considered “tool-deficient” in many aspects, mostly due to the lack of an effective, easy, inexpensive diagnostic test and the absence of a human vaccine. However, leptospirosis may be associated with poverty and to certain types of environmental or occupational risks. Therefore, developing both an early detection tool and a vaccine are crucial to saving lives.

## METHODS

The prioritization of health and research topics for the Rio meeting was based on the nominal group technique. The meeting attendees consisted of 20 decision makers (composed of 9 country representatives, 6 participants from other positions such as veterinary doctor or biologist working at the government not in managerial position and 5 representatives from international organizations); 20 researchers in leadership positions at their respective institutions; 2 postgraduate students with data relevant to the meeting; and 8 administrative-staff members from FIOCRUZ and the Pan American Health Organization (PAHO).

PAHO had invited 10 countries from Latin America to the meeting, and 9 of them sent a national representative responsible for leptospirosis to the event: Argentina, Brazil, Colombia, Costa Rica, Cuba, Honduras, Mexico, Panama, and Peru. The countries had been selected based on the reported number of cases, cumulative leptospirosis incidence rates and alerts, and regional representation. Before the meeting, PAHO had sent a survey to the country representatives, asking for more disaggregated epidemiological information and for answers to a questionnaire designed to identify country needs for surveillance and other key concerns, as well their views about possible solutions involving research, technological development, and innovation (RTDI) (Supplementary File 1).

PAHO combined the countries’ responses and prepared summaries to be presented at the beginning of each meeting session. Although those responses were all from Latin America, the presence of experts from other world regions at the meeting made it possible to identify shared needs and challenges.

The authors of this article obtained additional baseline data about the leptospirosis epidemiological situation in the different countries from the experts’ presentations at the Rio meeting. This information included the first set of regional leptospirosis data from the PAHO core indicators database as well as the responses to the PAHO questionnaires, which seven of the nine national representatives completed and returned.

After the Rio meeting, the authors drew up the road map and the recommendations, using the information from the event, including papers, slides, and recorded oral presentations and discussions, as well as a search for other, relevant scientific literature. The criteria for analysis, prioritization, and recommendations for health issues and RTDI included: 1) the magnitude of the problem in Latin America and in other areas of the world; 2) the impact on human and animal health, considering the One Health approach; and 3) the probability that the current technology and available resources could change the present situation.

## RESULTS

### Prediction

#### Leptospirosis as an emerging and neglected disease

During the meeting, recent studies were presented on the global public health burden of leptospirosis. It is estimated that every year over 1 million cases and 59 000 deaths occur due to the disease (11). Males account for approximately 80% of the total burden. A study by Torgerson and colleagues (15) found that the greatest burden is in the poorest countries in tropical regions. That study also predicted a high burden in regions such as Africa, where only limited data are available. In addition, those researchers estimated that, globally, nearly 2.90 million disability-adjusted life years (DALYs) are lost each year (with 2.80 million years of life lost due to mortality, and over 100 000 years lived with a disability) (15).

For most of the countries and territories of Latin America and the Caribbean that are located in tropical and subtropical areas, leptospirosis is emerging as a public health problem. A study conducted using information from HealthMap (a global database that utilizes various online sources for real-time surveillance of emerging public health threats) found that 63% of the global alerts on leptospirosis between the years 2007 and 2013 were reported in the Region of the Americas (12). However, comprehensive data on leptospirosis cases are also limited in the Americas. In 2015, PAHO included human leptospirosis in the Regional Core Health Data Initiative (16), and information from that source was presented during the Rio meeting. A journal article (17) on the first year’s data indicated that more than 10 000 human cases were reported in the Region of the Americas.

A systematic review of the epidemiology of leptospirosis in Africa (18) showed that while human disease data are lacking from many countries, there is evidence that leptospirosis is an important cause of febrile disease in that continent. The prevalence of leptospirosis among acute febrile patients ranged from 2.3% to 19.8%. Based on information from several studies, it was estimated that for the entire continent of Africa, the caseload could be up to 750 000 leptospirosis cases per year. It was also concluded that in Africa leptospirosis is endemic in both wild and domestic animals.

Leptospirosis is currently not included in the World Organisation for Animal Health (OIE) list of terrestrial animal diseases under surveillance. A geographic distribution and mapping of outbreaks in the Americas (19) showed that the disease is widely disseminated. Tropical terrestrial biomes are the predominant ecosystems associated with animal leptospirosis outbreaks. Several animal species (synanthropic rodents, domestic livestock, and wild animals) have serological evidence of infection or are carriers for *Leptospira*. The morbidity and the health impact associated with leptospirosis in livestock are expected to cause economic losses, but there are insufficient studies to properly quantify those costs.

#### Knowing where and when the risk is higher

As with many others infectious diseases, leptospirosis displays patterns in its spatial and temporal distribution that are driven by a complex web of socioecological factors. The goal of epidemiological and predictive mathematical modeling is to build knowledge about the mechanisms behind the observed patterns of infection. That research can improve our understanding of the occurrence of leptospirosis outbreaks and also identify high infection risk areas. A study using an ecosystem approach in the state of Rio Grande do Sul, Brazil, was described in one of the Rio meeting presentations. That research found the areas with the highest incidences of infection and also identified a set of environmental factors as the main predictors of disease—information that can be used to support better interventions (20). Another Rio presentation concerned a portfolio management model being developed to compare interventions for leptospirosis and to identify optimal choices under different conditions, by using complex system analysis and computational technology.

### Detection

#### Current tools and emerging technologies for leptospirosis diagnosis.

Conventional laboratory diagnostic tests that are currently used as global standards and references have many drawbacks. These shortcomings include delayed results, unreliability, a low detection threshold, difficult standardization, and requiring well-trained personnel and/or expensive media and equipment. The microscopic agglutination test (MAT) is considered to be the gold standard for serological diagnosis. However, it has only 80% sensitivity, and the confirmation of clinical suspicion may be late for clinical management. Due to the tedious growth of *Leptospira,* isolation using culture is slow and sensitivity is lower than with serological tests. The identification of *Leptospira* at the species and serovar levels is important for epidemiological knowledge, but the tools and expertise are restricted to a few reference laboratories around the world.

The rapid diagnostic tests currently available usually have low diagnostic accuracy and thus require confirmation by conventional tests. Techniques based on nucleic acid amplification may provide early diagnosis since they are more sensitive than isolation in culture media. Diagnostic tests to be developed in the next 5 to 10 years should be easy to perform, should provide early diagnosis, and should use portable devices for nucleic acid amplification or genome sequencing, in order to provide digital data and online transfer to clinicians and to surveillance systems.

It should be emphasized that the goals regarding the One Health approach may not be reached unless there are appropriate laboratory tools. One meeting presentation gave the example of laboratory-based surveillance with algorithms built for proper sample collection and laboratory tests currently used (both commercially available and complementary ones) applied in the surveillance system in Brazil. A total of 24 057 reported cases and 2 023 deaths in the 2010-2015 period were confirmed by clinical and laboratorial criteria. In another Rio presentation related to the One Health approach, ongoing studies about leptospirosis diagnosis in animals in the Caribbean country of Saint Kitts and Nevis were highlighted.

It should be noted that leptospirosis can occur concomitantly with other diseases that need to be considered in the differential diagnosis during outbreaks or periods of increased incidence of dengue and dengue hemorrhagic fever, yellow fever, hantavirus, and hantavirus pulmonary syndrome. Clusters of leptospirosis cases and deaths from febrile hemorrhagic diseases occurring mainly in rural areas often go undiagnosed.

#### Leptospirosis surveillance in selected countries of Latin America.

Of the nine Latin American countries with a national representative at the Rio meeting, seven of the nations had answered the PAHO survey. All seven of them include human leptospirosis as a disease of compulsory notification in their official surveillance systems. The internal flow of information commonly consists of daily alerts, with case reports sent to the central level and to laboratory for confirmation, according to each country’s guidelines. Some countries have established a committee to review the case reports and reach a final decision on them. Surveillance is an important component of the everyday activities under their national prevention and control programs.

At the Rio event, representatives from five countries described their epidemiological situation and surveillance system: Brazil, Colombia, Cuba, Honduras, and Peru. Table 1 summarizes that information.

**TABLE 1.** Reported leptospirosis cases and cumulative incidence in five Latin American countries, 2014

Country	Reported cases	Incidence per 100 000 population	Epidemiological aspects
Brazil	3 974	2.0	Leptospirosis is endemic in Brazil throughout the year. In 2014, large outbreaks were mainly detected after floods, and 84% of the cases occurred in urban areas. Brazil has a strategy to tackle the disease by defining priority municipalities based on several indicators, such as incidence, mortality, response capacity, and reference hospitals for patient care.
Colombia	867	1.8	In Colombia, leptospirosis outbreaks are most common during the fourth quarter of the year, which is when the greatest number of floods is expected. In 2014, 66% of the cases came from five departments (the first subnational administrative level) located in the mountainous Andean region.
Cuba	175	1.6	Leptospirosis is endemic in Cuba, with a potential for outbreaks. There has been a marked decrease in the number of cases since 1994, when a high number of human cases occurred and an emergency plan was developed that included the distribution of a nationally produced vaccine to at-risk groups.
Honduras	22	0.3	Leptospirosis is among the nine diseases considered under a national strategic plan for battling neglected tropical diseases. In 2014, two departments in the eastern part of the country had the highest number of cases, and only a few cases were reported in the western part of the country. A majority of cases (55%) occurred in rural areas.
Peru	2 329	7.6	Leptospirosis in Peru is mainly limited to forested areas in the eastern part of the country. One of the areas most affected by outbreaks is the city of Iquitos (region of Loreto), due to floods linked to the Amazon River overflow. The incidence in Loreto increased from under 1.0 per 100 000 population in 2012 to over 6.0 per 100 000 in 2014.

***Source***: Information presented by representatives from the five countries at the Rio de Janeiro November 2015 meeting.

### Prevention

#### Leptospirosis human vaccines.

The rationale for developing vaccines for human and animal use is based on the health and economic impact of the disease and the lack of effective prevention and control measures, especially in rural areas. There are many vaccines used in veterinary practice, and there are two vaccines licensed for human use (from Cuba and France). However, the available vaccines are preparations of the whole bacterial cell. Protective immunity appears to be associated with anti-lipopolysaccharide (anti-LPS) antibodies. The four main limitations of the available vaccines for human use are: 1) the lack of robust evidence for their effectiveness; 2) adverse reactions that reduce the acceptability for human use; 3) short-term immunity; and 4) multivalent formulations that may not be appropriate for different epidemiological situations, since immunity is serovar-specific (21).

The large number of serovars and species implicated in the etiology of leptospirosis has represented the greatest challenge to the development of vaccines. Recent efforts have focused on identifying candidates for subunit vaccines utilizing the high-throughput approaches currently available and applied to the development of new vaccines for several other bacterial pathogens. Such subunits of *Leptospira* as outer membrane proteins, virulence factors, and mediators of cellular interactions between pathogens and their natural hosts have been proposed. However, mediated immunity by vaccination, which provides cross-protection between serovars, is a goal to be achieved (22, 23).

Filling some knowledge gaps is important for developing and applying vaccines. Experimental models for proof of concept are based on death as the end point. There are limitations regarding the correlation between the model and the natural infection in humans or domestic animals. Evidence of natural immunity is indirect and currently consists of the detection of agglutinating antibodies. Key actions that are needed to meet the goal of having a new-generation vaccine were presented at the Rio meeting. These included carrying out more and more-targeted research, building partnerships between key stakeholders, conducting training at different levels, and encouraging investment.

#### Studies and interventions in animals.

Studies and interventions in animals comprise an extensive field for research and actions related to health policy. It is known that rodent control is important for reducing the number of human cases. At the Rio event, representatives from Brazil presented the example of an integrated approach for rodent control. It includes education, training of health professionals, surveillance, risk stratification, control measures, and eco-epidemiological studies. This approach reduced rat infestation from 25% to 7% of households. Studies carried out in the African country of Niger that included trapping rodents and testing for leptospirosis found peculiar transmission patterns for the disease. Those patterns were mainly associated with urban gardens, calling attention to unexpected eco-epidemiological situations (24).

The presentation from New Zealand during the Rio meeting included mention of the fact that leptospirosis had been recognized as an occupational problem there in the 1970s (when there was high incidence among dairy workers), and also later in the pig industry. Currently, the incidence of leptospirosis in humans in New Zealand is about 2.0 to 2.5 per 100 000, which is relatively high compared to other high-income countries. These cases are predominantly associated with agriculture, on which New Zealand is highly dependent economically, particularly the dairy, sheep, deer, and beef industries. Occupational infection is most common among meat industry workers and farmers. One measure for leptospirosis control that has been beneficial is the use of personal protection in farms. Another is vaccination in cattle, which has been shown to reduce shedding. Animal vaccination for leptospirosis in dairy farms has been commonplace in New Zealand for the past 40 years, and it is likely the most effective control measure.

### Response

#### Human case management.

At the Rio meeting, the experiences of Brazil, Peru, and Thailand in both endemic and epidemic situations were presented, with a focus on the challenges of medical practice. In case management in Thailand, a wide clinical spectrum of disease severity has been found. Human leptospirosis ranges in severity from a mild, self-limited febrile illness to a fulminant, life-threatening one. Most cases are uncomplicated, anicteric disease, with around 10 days of fever and a mortality of less than 1%. Uncomplicated disease is a major cause of morbidity, but rapid, reliable laboratory diagnosis is not widely available. In addition, the mild forms may be misdiagnosed because the clinical presentations are similar to those for several other infectious diseases.

It is estimated that 10% to 15% of leptospirosis cases show the most severe manifestations of the disease, known as Weil’s syndrome (jaundice-hemorrhage-renal failure), and are more likely to be recognized as leptospirosis based on signs and symptoms. This syndrome is an important cause of death, with a case-fatality rate of 10% to 12%. However, severe pulmonary forms of leptospirosis have been described in various geographic areas. These severe pulmonary forms are characterized by massive pulmonary hemorrhage and renal failure, with case-fatality rates of 50% to 70%. These forms have been given different designations, including adult respiratory distress syndrome (ARDS), severe pulmonary form of leptospirosis (SPFL), and pulmonary hemorrhage syndrome associated with leptospirosis (PHSL) (1, 25). According to a Rio meeting presentation, these challenges for clinical management in hospital intensive care units (ICUs) have been seen in the city of São Paulo, Brazil.

A critical point is to evaluate the prognostic factors of severe forms that require hospitalization. This is particularly important in emergency situations such as outbreaks associated with weather changes and flooding. Some of the Rio meeting participants suggested that the guidelines for clinical management prepared by experts in Brazil be made available to other countries. These guidelines include epidemiological criteria related to risk exposure in natural disasters; anamnesis and physical examination with chronology of signs and symptoms; warning signs and indications for hospital admission; diagnostic procedures with algorithms; clinical management in intensive care units; clinical support and antibiotic therapy; and hospital discharge criteria (26).

### Fundamental knowledge gaps and challenges for research, technological development, and innovation

The main scientific knowledge gaps for the road map for leptospirosis are related to the prediction, detection, prevention, and clinical management of human cases. Challenges from the scientific point of view were presented by several Rio meeting participants. Aspects of RTDI were framed within the concepts of One Health and translational research. The presentations covered important aspects of biomedical research, emphasizing the etiological agents and their hosts.

Three meeting presentations illustrated possibilities for further developments, including whole genome applications for diagnosis and vaccines, as well as experimental models to understand the molecular mechanisms of leptospirosis pathogenesis. One presentation looked at how and why to type the agents of leptospirosis. The second presentation considered the genome of *Leptospira interrogans* serovar Copenhageni and the identification of potential targets for diagnosis and vaccines. The third examined the significance of understanding the relationship between leptospires and their reservoir hosts of infection.

Results from studies carried out in French overseas departments in the Caribbean (Guadeloupe and Martinique) and in the Indian Ocean (Mayotte) (27) were presented at the Rio meeting. Data from more than 150 isolates from Guadeloupe and Martinique and more than 200 from Mayotte have identified new pathogens by serological and/or molecular techniques at the species level (*Leptospira mayottensis*), serovars (Tabaquite, Bajan, and unknown serovars), new genotypes, and sequence types. These data illustrate the biodiversity of *Leptospira*. Perspectives in this field should take into account the identification of the etiological agent by next-generation sequencing, RNA-based PCR assays, and the development of LPS-based molecular methods (27-29).

The pan-genome analysis of *Leptospira* species remains a key gap in identifying potential targets for diagnosis and in generating new vaccines (30). Results from several studies using *L. interrogans* serovar Copenhageni to explore genome sequences in search for candidates for diagnostic tests and vaccines and for a better understanding of pathogenesis were presented in Rio. Those studies covered: 1) the availability of the whole genome sequence of Copenhageni; 2) *in silico* analysis for protein selection; 3) the pipeline to obtain recombinant proteins; and 4) identification of novel extracellular membrane (ECM) binding proteins with a possible role in pathogenesis through adhesion to eukaryotic cells and their possible relations with molecular mechanisms of pathogenesis. Putative novel proteins and mechanisms of cell invasion possibly associated with adhesins that have been described in previous publications were also covered at the Rio meeting. The construction of chimeric proteins based on B/T lymphocytes epitopes or known immunoprotective regions is considered to be a promising strategy for vaccine candidates (31).

The biodiversity of leptospires is likely to be associated with a large diversity of animal hosts in the mammal clade. Rodents are the universal reservoirs, while man is an accidental host, in which the infection may be lethal. Results obtained from experimental infection in rodents have been key for understanding the relationships between *Leptospira* and the animal hosts, according to data from recent publications that were covered during the meeting. The main findings dealt with different patterns of renal tissue changes and distribution of antigens, with modulation of gene and protein expression by leptospires in response to mammalian host signs. Understanding the mechanisms involved in leptospirosis pathogenesis and immunity is crucial for new therapeutic alternatives and vaccines. Information about gene and protein expression modulation by leptospires in response to mammalian host signals represents a step forward for further developments (32, 33).

## ROAD MAP

Table 2 summarizes the leptospirosis road map and encompasses the report of countries’ needs by relevant topic according to the survey, identified scientific challenges, ongoing research to address those needs, and recommendations. The health policy recommendations should be taken into account by decisionmakers, government officials, and PAHO/WHO. The priorities for research, technological development, and innovation (RTDI) should be considered by research institutions, universities, and stakeholders. The list of general recommendations is presented in Supplementary File 2.

**TABLE 2.** Summary of road map for leptospirosis research and health policies in Latin America

Report of countries’ needs	State of the art	Recommendations for decision makers and PAHO/WHO	Priorities for research, technological development, and innovation (RTDI)
Surveillance	- Availability of diagnostic tests - Capacity-building for clinical and laboratorial diagnosis - Strengthening surveillance for human and animal leptospirosis and for environmental contamination	- Many countries in Latin America with surveillance systems in place ready to recognize human and animal leptospirosis - Leptospirosis is notifiable in many countries of Latin America - Laboratory confirmation is usually based on conventional serological tests - Isolation and identification of *Leptospira* is rare - PCR-based techniques are usually performed in-house in a few central laboratories	- Provide guidelines for leptospirosis surveillance and information materials - Put forward training programs on clinical and laboratory diagnosis - Improve national surveillance systems to increase the geographical coverage of leptospirosis data in Latin America - Monitor outbreaks - Evaluate criteria for case definition and laboratory confirmation - Adopt prevention and control measures - Develop a collaborative data-sharing program to integrate human-animal-environment interface	- New, affordable diagnostic tests to support laboratory-based surveillance - Accurate quantification of human and animal leptospirosis cases - Geographic distribution of *Leptospira* species and serovars, animal carriers, and human cases
Prediction	- Software to integrate different types of databases for risk stratification, prediction, and forecasting - Training for personnel in appropriate quantitative methods - Integration of multidisciplinary research teams - Exchange of timely information among sectors	- An increasing number of scientific groups are working on leptospirosis prediction, and several studies including modeling have been published - Country surveillance data could be used for epidemiological analysis and prediction - Several countries have epidemiological information, but it is usually not open access - There are some open-access environmental and socioeconomic databases that can be integrated into surveillance case data	- Description and analysis of country case data should be done at different scales - Improve and standardize methodology to produce the needed epidemiological information for predictive modeling - Support the identification of risk areas and risk groups - Training in outbreak detection and response - Training in quantitative methods for data management and analysis - Support research that shares surveillance data for prediction	- Understand the transmission patterns at the community level - Develop prediction tools to support countries for early warning of possible outbreaks - Partnership among researchers and health authorities in information exchange and use of the prediction models - Define set of variables (possible drivers) to explain outbreaks and higher incidence, in order to support interventions - Large-scale spatial-temporal modeling for early warning system for leptospirosis outbreaks - Modeling effectiveness and impact of chemoprophylaxis as an intervention for infection risk reduction - Model to evaluate vaccine interventions based on occupational risk and geographical risk areas
Diagnosis	- Availability of tests, reagents, and reference strains for case confirmation - Establish or strengthen national laboratory networks - Training of personnel in new methods of diagnosis	- Available diagnostic tools may not be enough for timely aid in clinical management and may be of limited support for epidemiological surveillance	- Commercial tests must be validated on-site before being recommended by surveillance systems - Establish and use algorithms for proper diagnosis according to set times for detection of antigens, amplified nucleic acids, and antibodies	- New developments on nucleic acid amplification–based techniques for early diagnosis - Rapid diagnostic tests (RDTs) for point-of-care diagnostic testing - Improvement of current systems for identification of clinical isolates
Human clinical management	- Training of physicians on clinical diagnosis and treatment - Including leptospirosis in medical students’ clinical program - Updated available guidelines - Research on new treatments - Educational materials	- Misdiagnosis with acute febrile diseases and hemorrhagic febrile diseases - Severe forms of leptospirosis still represent a challenge in clinical practice, with high case-fatality rates - A major issue is the clinical management of the pulmonary hemorrhage syndrome associated with leptospirosis (PHSL)	- Provide guidelines for leptospirosis clinical management - Design and deliver training programs for leptospirosis recognition and clinical care - Support researchers to identify clinical isolates	- Tests for early diagnosis and timely aid in clinical treatment - Clinical studies to provide scientific evidence for clinical management of severe forms - New therapeutic alternatives to reduce case-fatality rates
Vaccines	Vaccine (if available) could be used in the following occupational groups: agricultural workers, animal handlers, sewage and water supply workers, veterinarians, and the military	- Only one country in the Americas uses human vaccine on a regular basis - Current vaccines for human and veterinary use are whole-cell vaccines - There are two vaccines licensed for human use - Limitations and controversies are mainly related to the formulations (immunity is serovar- specific) and the lack of robust evidence from randomized controlled trials (RCTs) - Recent efforts have been largely focused on subunit vaccine candidates	- Evaluating the recommendation of vaccines licensed for human use to prevent leptospirosis	- Studies about candidates for subunit vaccines, natural immunity, experimental models for proof of concept, and target populations to be vaccinated
Studies and interventions in animals	-Training technical health and agriculture personnel in rodent control methods - Guidelines and methodologies for rodent control - Conduct studies on rodents and other animal reservoirs	- Animal vaccines available in most of the countries but not always with official quality control - Animal vaccination for leptospirosis in dairy farms has been successful in other world regions - Many countries in Latin America (especially Brazil) have experience in rodent control, but this knowledge needs to be shared with other countries - Criteria for risk areas used for rodent control in São Paulo	- Provide guidelines for rodent control in urban and rural settings - Hold training on rodent control - Use of personal protection for workers at risk - Coordinate partnerships between public health and agriculture to meet goals of surveillance and control for human and animal leptospirosis, considering the One Health perspective	- Studies on new vaccines for livestock to prevent the disease and the carrier state - Studies about dynamics of rodent populations and human infection
RTDI	- Simple tests for diagnosis - Identification of the etiological agent, animal carriers, and risk areas - Studies about incidence, prevalence, and case-fatality rate in humans	- Knowledge gaps on leptospirosis biomedical research must be considered globally - Only new and cutting-edge technologies could bring about the required progress on diagnosis, vaccines, and therapeutic alternatives - Epidemiological and clinical studies are essential to leverage and support other fundamental research areas (molecular pathogenesis, immunology, genome, proteome, and related fields)	- Stimulate operational research to support the needs related to surveillance systems	- Biodiversity and geographic distribution of *Leptospira* spp. and serovars - Application of new tools for development of diagnostic tests, vaccines, and therapeutic alternatives - Experimental models to better understand the leptospirosis pathogenesis and to support new developments on vaccines and therapeutic alternatives

***Source***: Developed by the authors, based on information presented at the Rio de Janeiro November 2015 meeting. as well as other, relevant scientific literature.

## CONCLUSIONS

The conclusions listed below were reached by consensus in the group discussions at the Rio meeting:

1. Leptospirosis is not considered to be a “tool-ready” disease for global initiatives. Surveillance programs are not equipped with the necessary tools for early detection, prevention, and follow-up strategies. This is despite the wide geographic distribution of leptospirosis and its impact in terms of incidence, morbidity, and mortality.
2. The scenario presented by data from the Americas, Europe, Asia, Africa, and New Zealand corroborates the complexity of transmission cycles, which involve a range of animal carriers, several species and serovars as etiological agents, and different environmental factors. This complexity presents biological, environmental, and scientific challenges.
3. The One Health approach should be considered in addressing further developments and initiatives for prevention, control, and the ultimate goal of saving lives.
4. There are technical and scientific knowledge gaps that hinder epidemiological surveillance and that cut across leptospirosis clinical management, prevention, and control.
5. Identified knowledge gaps should be addressed at different levels of health policies and supported by new scientific developments.

## Acknowledgments

We thank all the Rio meeting participants, whose contributions made it possible to develop the road map for leptospirosis research and health policies in Latin America. In particular, we would like to recognize all the members of the organizing and scientific committees and the country representatives for their valuable support: A. Ko, C. Munoz-Zanzi, C. Schneider, D. Galan, E. Bertherat, E. Caldas, E. Vazquez, F. Costa, F. Mendigaña Paez, F. Pinna, H. Cedeño, J. Arrebato, J. Barbosa, J. Benschop, J. Nally, J. Navarro, J. Martinez, L. Contretas, L. Geffner, L. Fonseca, M. Jancloes, M. Pereira, M. Picardeau, O. Cosivi, P. Buss, R. Hartskeerl, R. Lima, R. Velasquez, S. Aldighieri, S. Nishioka, S. Valéria, W. Savino, and Y. Salazar.

## Funding.

Sponsorship and support for the meeting came from the Ministry of Health of Brazil (Oswaldo Cruz Institute/FIOCRUZ, the Secretariat of Health Surveillance, and the Secretariat of Science and Technology), the Pan American Health Organization (PAHO/WHO), and GLEAN.

## Disclaimer.

Authors hold sole responsibility for the views expressed in the manuscript, which may not necessarily reflect the opinion or policy of the *RPSP/PAJPH* or PAHO.
